# Rheumatoid Arthritis

**Published:** 1874-01-15

**Authors:** 

**Affiliations:** Pennsylvania Hospital


### RHEUMATOID ARTHRITIS.

### CASES FROM CLINIC OF PROFESSOR DA COSTA, IN PENNSYLVANIA HOSPITAL.

*From Philadelphia Medical Times.*

THE affection which we are about to consider has been known by various names, such as rheumatic gout, rheumatoid arthritis, etc., which latter has been generally adopted as a sort of compromise between the two prevailing and contending theories as to its nature — rheumatic or gouty. It affects persons of an anæmic or scrofulous constitution, and is usually traceable to exposure to cold or damp. It begins with slight swelling of the smaller joints, as a rule, with tenderness, but no discoloration. The swellIng is due to an inflammation, with effusion of water or pus, which finally subsides, and the swelling disappears. This is now followed by a thickening of the synovial membranes, and the formation of "vegetations," which gradually harden, and stiffness of the joint supervenes.

Second attacks are apt to follow upon partial convalescence, leading to complete disorganization of the joints affected; finally, we find loss of articular cartilage, the bones, becoming eburnated, produce the peculiar grating sound noticed when the joints are moved. Dislocation is a frequent sequence.

The constitutional involvement is peculiar, and, as we become more familiar with the disease, affords a valuable aid in its diagnosis. It is usually subacute, and presents no fever-phenomena, no uric acid or increase of fibrin in the blood, and no acid perspiration; neither are there deposits in the finger-joints and ears, as in gout. There is no history of hereditary rheumatism or gout. The absence of cardiac symptoms is peculiar. These lesions are so intimately associated with rheumatism as to warrant the assertion that four-fifths of the cardiac diseases are attributable to rheumatism. These points in the diagnosis and clinical history are proofs of its being a *distinct disease.*

Appended is the clinical history of two cases in point:

CASE I.—A man, aged thirty years, a shoemaker by trade, first became affected, two years since, with swelling, pain, and stiffness in the great toe of the left foot, which have extended to all of the larger joints. He gave, upon admission, a history of previous good health; never had fever during the progress of his disease; never had syphilis; and there is no family history of gout or rheumatism. It came on gradually. His urine is normal, bowels regular, and he has a good appetite. He has lost flesh, but is now gaining again. The hands present the peculiar distorted appearance of the disease, and there is "grating" upon motion; the large joints are alike affected, being rigid and painful upon motion. Auscultation reveals, at the base of the heart, a soft, systolic murmur, which is not constant; there is no hypertrophy. He has been taken citrate of lythia and cod-liver oil; this, conjoined with baths and regulated diet, has produced favorable results.

CASE II.—A French sailor, who has been in the house five days, states that he has been affected two months, giving no acute history; there is some stiffness of the smaller joints; entire absence of heart-lesions; no fever, tongue somewhat coated; pulse and temperature normal.

This patient might take, with advantage, lithia, cod-liver oil, or arsenic, internally, while he is kept at rest in bed, and leeches applied; then cold water or lead-water and laudanum applied locally. Frequently diuretics, and occasional purging, are useful in the early stages of the disease. If there is much weakness, quinia is given with advantage.

Later in the disease, the local indications require more urgent treatment, and here iodine may be used freely, or better, the following:

℞.—Potass. iodid., ʒ ij.

Lin. sapon. camph., f ℥ vj.

Tr. belladonnæ, f ℥ ij.

To be applied morning and evening.

Ammoniacal and mercurial plaster may serve a good purpose.

Internally, cod-liver oil, potass. iodid., liq. potass. arsenit., iodide of iron, and citrate of lithia, are the remedies *par excellence.* A formula now used in the hospital is:

℞.—Effervescing citrate of lythia, gr. iij. to v.
Cod-liver oil, f ℥ ss.

Arsenic is valuable; in fact, no case should be pronounced incurable until it has been tried. If the nutrition fails, the system may be supported with stimulants, as there is no contra-indication, as in gout or rheumatism. They are not to be used in the acute form of the disease, however.

Baths should be insisted upon; for this purpose, tepid water and carbonate of soda may be employed; or Turkish baths may be used. Finally, a change of climate may be of service.

